# Estimate of Venous Thromboembolism and Related-Deaths Attributable to the Use of Combined Oral Contraceptives in France

**DOI:** 10.1371/journal.pone.0093792

**Published:** 2014-04-21

**Authors:** Aurore Tricotel, Fanny Raguideau, Cédric Collin, Mahmoud Zureik

**Affiliations:** Department of Epidemiology of Health Products, French National Agency for Medicines and Health Products Safety (ANSM), Saint-Denis, France; Indian Institute of Toxicology Research, India

## Abstract

**Purpose:**

To estimate the number of venous thromboembolic events and related-premature mortality (including immediate in-hospital lethality) attributable to the use of combined oral contraceptives in women aged 15 to 49 years-old between 2000 and 2011 in France.

**Methods:**

French data on sales of combined oral contraceptives and on contraception behaviours from two national surveys conducted in 2000 and 2010 were combined to estimate the number of exposed women according to contraceptives generation and age. Absolute risk of first time venous thromboembolism in non-users of hormonal contraception and increased risk of thromboembolism in users vs. non-users of hormonal contraception were estimated on the basis of literature data. Finally, immediate in-hospital lethality due to pulmonary embolism and premature mortality due to recurrent venous thromboembolism were estimated from the French national database of hospitalisation and literature data.

**Results:**

In France, more than four million women are daily exposed to combined oral contraceptives. The mean annual number of venous thromboembolic events attributable to their use was 2,529 (778 associated to the use of first- and second-generation contraceptives and 1,751 to the use of third- and fourth-generation contraceptives), corresponding to 20 premature deaths (six with first- and second-generation contraceptives and fourteen with third- and fourth-generation contraceptives), of which there were eight to nine immediate in-hospital deaths. As compared to the use of first- and second-generation contraceptives, exposure to third- and fourth-generation contraceptives led to a mean annual excess of 1,167 venous thromboembolic events and nine premature deaths (including three immediate in-hospital deaths).

**Conclusions:**

Corrective actions should be considered to limit exposure to third- and fourth-generation contraceptives, and thus optimise the benefit-risk ratio of combined oral contraception.

## Introduction

Women using combined oral contraceptives are exposed to an increased risk of venous thromboembolism [1], [2] and, to a lesser extent, of arterial thromboembolism [3], [4]. Contrary to the risk of arterial thromboembolism, the risk of venous thromboembolism varies amongst combined oral contraceptives, depending on which type of progestogen they contain.

Since 1995, epidemiological studies have shown a higher risk of venous thromboembolism with combined oral contraceptives containing gestoden and desogestrel (so-called third-generation pills) compared to products containing levonorgestrel (so-called second-generation pills) [5]–[10]. A similar increased risk has been more recently observed in women using combined oral contraceptives containing drospirenone (so-called fourth-generation products) [10]–[12]. Pulmonary embolism, as a life-threatening condition, is the most serious manifestation of venous thromboembolism with significant mortality.

Although mentioned in the summary of product characteristics (SPCs) for combined oral contraceptives, venous and arterial thromboembolic risks are little-known to users and appear to be underestimated by healthcare professionals. As a result, there was an uproar in the French population when in December 2012 a young woman brought a lawsuit against both a pharmaceutical company and the French National Agency for Medicines and Health Products Safety (ANSM) after suffering a debilitating stroke while taking a third-generation contraceptive. Yet, considering that there was no scientific data supporting that third- and fourth-generation products differ from first- and second-generation contraceptives in terms of efficacy and tolerability, notably with regard to weight gain and acne, the ANSM and the French National Authority for Health (HAS) had already recommended that the most recent generations should not be used in a first-line indication.

As in other European countries, combined oral contraceptives are widely used in France. In 2011, approximately 4.3 million French women were exposed each day and half of them were using a third- or fourth-generation contraceptive [13], [14]. Taking into account the significant number of exposed women in Europe and the damaging effect of third- and fourth-generation contraceptives regarding venous thromboembolism and liable lethality due to pulmonary embolism, the ANSM, in January 2013, requested the European Medicines Agency (EMA) for a wide review of these products [15]. In this context, the ANSM has assessed the consequences of the large exposure to combined oral contraceptives at national level in terms of morbidity and mortality due to venous thromboembolism over a 12 years period.

Using a pharmacoepidemiological approach, this study aims to estimate the number of venous thromboembolic events and related-premature deaths due to pulmonary embolism (including immediate in-hospital lethality) attributable to the use of combined oral contraceptives in exposed women of childbearing age, between 2000 and 2011.

## Methods

Four steps were required to answer the study objectives: (1) the estimation of the annual number of women exposed to combined oral contraceptives by age and by generation, (2) the absolute risk of first time venous thromboembolism among users of combined oral contraceptives estimated from a population of non-users, (3) the combined oral contraceptives-associated relative risk (RR) of venous thromboembolism, (4) the premature mortality due to pulmonary embolism, including immediate in-hospital lethality.

### Existing data and working hypothesis

**(1) Exposure to combined oral contraceptives in France.** The available data that served as a basis for our estimations are detailed below:
  - Data on the French population census were used to determine the number of women of childbearing age (i.e. aged 15–49 years) by five year age groups from 2000 to 2011 [16].
  - Data on sales of combined oral contraceptives annually claimed to the ANSM by the marketing authorisation holders were used to estimate the mean annual number of women daily exposed over the period 2000–2011, overall and by generation (first-/second-generation versus third-/fourth-generation) [13], [14]. The classification of combined oral contraceptives according to progestogen type is provided in table 1.

**Table 1:** Classification of combined oral contraceptives according to the type of progestogen.

Generation	Type of progestogen
First-generation combined oral contraceptives	Norethisterone
Second-generation combined oral contraceptives	Levonorgestrel, Norgestrel
Third-generation combined oral contraceptives	Desogestrel, Gestodene, Norgestimate
Fourth-generation combined oral contraceptives	Chlormadinone, Drospirenone, Nomegestrol, Dienogest

- Data from two French cross-sectional surveys investigating contraception behaviours, Cocon and Fecond, were used to estimate the distribution of combined oral contraceptives users within five year age groups, together with the spreading of the generations of pills [17], [18]. Both studies were conducted in representative samples of women of childbearing age. The Cocon survey was performed in 2000 in 2863 women aged 18–44 years. In the Fecond survey conducted in 2010, 5275 women aged 15–49 years were investigated over the phone.

**(2) Absolute risk of first time venous thromboembolism.** Venous thromboembolism is strongly age-dependent, hence the necessity to use accurate data in the population of women of childbearing age. In France, there is no population-based venous thromboembolism register. The only available French incidence rates of venous thromboembolism were estimated in a community-based study including 342000 Western France inhabitants. The overall incidence for first events of deep venous thrombosis and pulmonary embolism was 42 per 100 000 women aged 20–39 years and 71 per 100 000 women aged 40–59 years [19]. Results of two international studies based on data from administrative health care databases showed similar estimated annual incidence rates, ranging from 11 per 100 000 women aged 15–19 years to 82 per 100 000 women aged 45–49 years [20], [21]. The most recent and powerful safety study that assessed the risk of a first ever venous thromboembolic events among users of hormonal contraception has permit to document absolute risk in non-users of all type of hormonal contraception (never users plus former users), used as the reference group for relative risks estimate [10]. With near 1.5 million women aged 15–49 years included and 10 millions observation years, the Danish historical registry cohort study reported incidence by five year age groups. After exclusion of pregnant women, bilaterally oophorectomised, hysterectomised or sterilised women, and women with previous cardiovascular disease including venous thromboembolism, malignant disease or coagulation disturbances, the absolute risk of first time venous thromboembolism in non-users was estimated to 37 per 100 000 women years, ranging from 7 per 100 000 women years aged 15–19 years to 58 per 100 000 women years aged 45–49 years.

Since there is currently no evidence that suggest any European geographic variation of venous thromboembolism, absolute risks from the Danish cohort were used as estimates for the calculation of the number of first time thromboembolic events in French women exposed to combined oral contraceptives (table 2). We assumed that these incidence rates were constant over 2000–2011.

**Table 2:** Incidence rate of venous thromboembolism in non-users of oral contraceptives per 100,000 women years [10].

Age group (years)	Incidence rate of venous thromboembolism per 100,000 women-year (95% confidence interval)
15–17 Y	0.07 (0.05–0.09)
18–19 Y	0.21 (0.16–0.26)
20–24 Y	0.29 (0.24–0.34)
25–29 Y	0.32 (0.28-0.36)
30–34 Y	0.35 (0.31–0.39)
35–39 Y	0.48 (0.44–0.52)
40–45 Y	0.58 (0.53–0.63)

**(3) Combined oral contraceptives-associated relative risk of venous thromboembolism.** Epidemiological studies suggested that, compared to non-users, the risk of venous thromboembolism was approximately doubled in users of levonorgestrel-containing combined oral contraceptives and roughly quadrupled in users of products containing desogestrel, gestodene and drospirenone [5]–[12]. The Danish study found those results [10]. As compared to non-pregnant non-users of hormonal contraception without any known risk factor of venous thromboembolism, the relative risk of a first time thromboembolic event, confirmed or not, in users of hormonal contraceptives containing 30–40 μg ethinylestradiol combined with norethisterone was 1.6 (95% confidence interval 0.8 to 2.9), with levonorgestrel was 2.2 (1.7 to 0.8), with norgestimate was 2.6 (2.2 to 3.0), with desogestrel was 4.2 (3.6 to 4.9), with gestodene was 4.2 (3.9 to 5.1) and with drospirenone was 4.5 (3.9 to 5.1). RR were slightly lower in users of third-generation contraceptives containing 20 μg ethinylestradiol: 3.3 (2.9 to 3.7) and 3.5 (3.1 to 4.0) with desogestrel and gestodene respectively. Moreover, desogestrel, gestodene and drospirenone associated with 30-40 μg ethinylestradiol, as compared to oral contraceptives containing the same dosage of ethinylestradiol and levonorgestrel, were found to confer at least twice the risk of confirmed venous thromboembolism (RR  =  2.2 [1.7–2.8], RR  =  2.1 [1.7–2.5], RR  =  2.1 [1.7–2.7], respectively).

Based on this literature review, in our study, we retained a twofold risk in users of combined oral contraceptives containing norethisterone or levonorgestrel and a fourfold risk in users of products containing desogestrel, gestodene or drospirenone (compared to non-users). An increased risk similar to the one retained for products containing desogestrel or gestodene was assumed for combined oral contraceptives containing norgestimate, chlormadinone, nomegestrol or dienogest, despite the lack of literature data.

**(4) Pulmonary embolism.** Pulmonary embolism represents about one third of thromboembolic events [10], [19], [21]–[23]. We assumed that this rate was equally distributed within each five year age group and remained constant over the study period.
**(5) Premature mortality due to pulmonary embolism.** We restricted our estimations to premature mortality due to pulmonary embolism defined as mortality within five years due to recurrent pulmonary embolism. This definition includes immediate in-hospital lethality due to pulmonary embolism, i.e. mortality occurring during the course of the hospitalisation for pulmonary embolism.

Immediate in-hospital lethality due to pulmonary embolism was estimated in non-pregnant women aged 15–49 years without history of malignancy from the French national hospitalisation discharges database, in which all admissions in private and public hospitals are recorded [24]. From 2007 to 2011, the mean in-hospital lethality rate was 1.0%: 0.6% (95% confidence interval: 0.4 to 0.8%) in women aged 15–34 years and 1.3% (95% confidence interval: 1.1 to 1.6%) in those aged 35–49 years. These mean rates, assumed to be constant over time, were applied to the period 2000–2006.

Short-, mid- and long-term mortality due to pulmonary embolism is strongly age-dependent. From the rare published studies assessing predictive factors for fatal pulmonary embolism, mortality rates are often reported in subjects aged greater than fifty and are not stratified by age [25], [26]. Nevertheless, case-fatality rate due to pulmonary embolism after a first-time idiopathic pulmonary embolism was assessed among both a young and healthy population in a Californian study [27]. Conducted in 3459 patients aged 18 to 55 years of age (mean age of 41.9 years), the study reported a case-fatality rate due to pulmonary embolism within five years of 2.3%, corresponding to 1.6% within the first six months then 0.16% per year. In our study, we used the case-fatality rate of the Californian study on the assumption that the rate was constant over the study period 2000–2011 (2.3% [1.8 to 2.8%]).

### Estimations

Calculation made to estimate the annual number of venous thromboembolism in exposed women is detailed as follow:

**(1) Annual number of women exposed to combined oral contraceptives.** *Overall exposure*. Mean annual number of women daily exposed to a combined oral contraceptive was estimated by dividing the annual number of blister packs claimed to the ANSM by 13, i.e. the maximum number of blisters used per woman and per year.

*Exposure by age and by generation*. Trends in sales data from ANSM and results from the national surveys Cocon and Fecond were combined to estimate the annual utilisation rates of combined oral contraceptives by five year age group (i.e. 15–19, 20–24, 25–29, 30–34, 35–39, 40–44 and 45–49 years) and by generation. The number of women annually exposed to a combined oral contraceptive was obtained by applying these estimated utilisation rates to the number of French women of the considered age group (from data on French population census), and then adjusted with the estimation of the mean number of exposed women from sales. The calculation methodology is detailed in appendix S1.

**(2) Annual number of venous thromboembolism in exposed women.** The annual number of venous thromboembolic events occurring in users of combined oral contraceptives was the sum of the number of cases within each five year age group, estimated by multiplying the number of women exposed to a given generation by the corresponding RR (compared to non–use), i.e. twice and four times the absolute risk of first time venous thromboembolism for first-/second-generation and third-/fourth-generation, respectively.
**(3) Annual number of venous thromboembolism and pulmonary embolism attributable to the use of combined oral contraceptives.** Attributable fractions corresponding to retained RR were used to calculate the annual number of venous thromboembolic events that can be attributed to the use of combined oral contraceptives, by generation (compared to non-use) and between generations (i.e. first-/second-generation versus third-/fourth-generation). Attributable fractions were obtained using the following formula: (RR-1)/RR, where RR is the relative risk associated to the use of first-/second-generation or third-/fourth-generation.

The annual number of venous thromboembolic events attributable to the use of first-/second- and third-/fourth-generation products (compared to non-users) was estimated by multiplying the associated product-attributable fraction (i.e. 50% and 75%, respectively) by the annual number of venous thromboembolism occurring in women exposed to the concerned generation.

The annual number of venous thromboembolism attributable to the use of third-/fourth-generations (compared to the first-/second-generations) was estimated by multiplying the third-/fourth-generation-attributable fraction, i.e. 75%, by the annual number of venous thromboembolism occurring in women using these products.

For all calculations, the number of pulmonary embolisms attributable to the use of combined oral contraceptives was obtained by dividing the number of venous thromboembolism attributable by three.

**(4) Sensitivity analyses.** Five sensitivity analyses were conducted. The first analysis was performed by varying combined oral contraceptives-associated relative risk of venous thromboembolism with the extreme values the most frequently founded in the literature. The next three analyses were conducted by using lower and upper limits of 95% confidence intervals of the rates used to estimate the number of venous thromboembolic events or related-deaths: incidence rates of venous thromboembolism from the Danish study, immediate in-hospital lethality rates from French hospitalisation data and premature mortality rate from the Californian study. A last sensitivity analysis was performed combining all previous sensitivity analyses.

## Results

**Figure 1:**
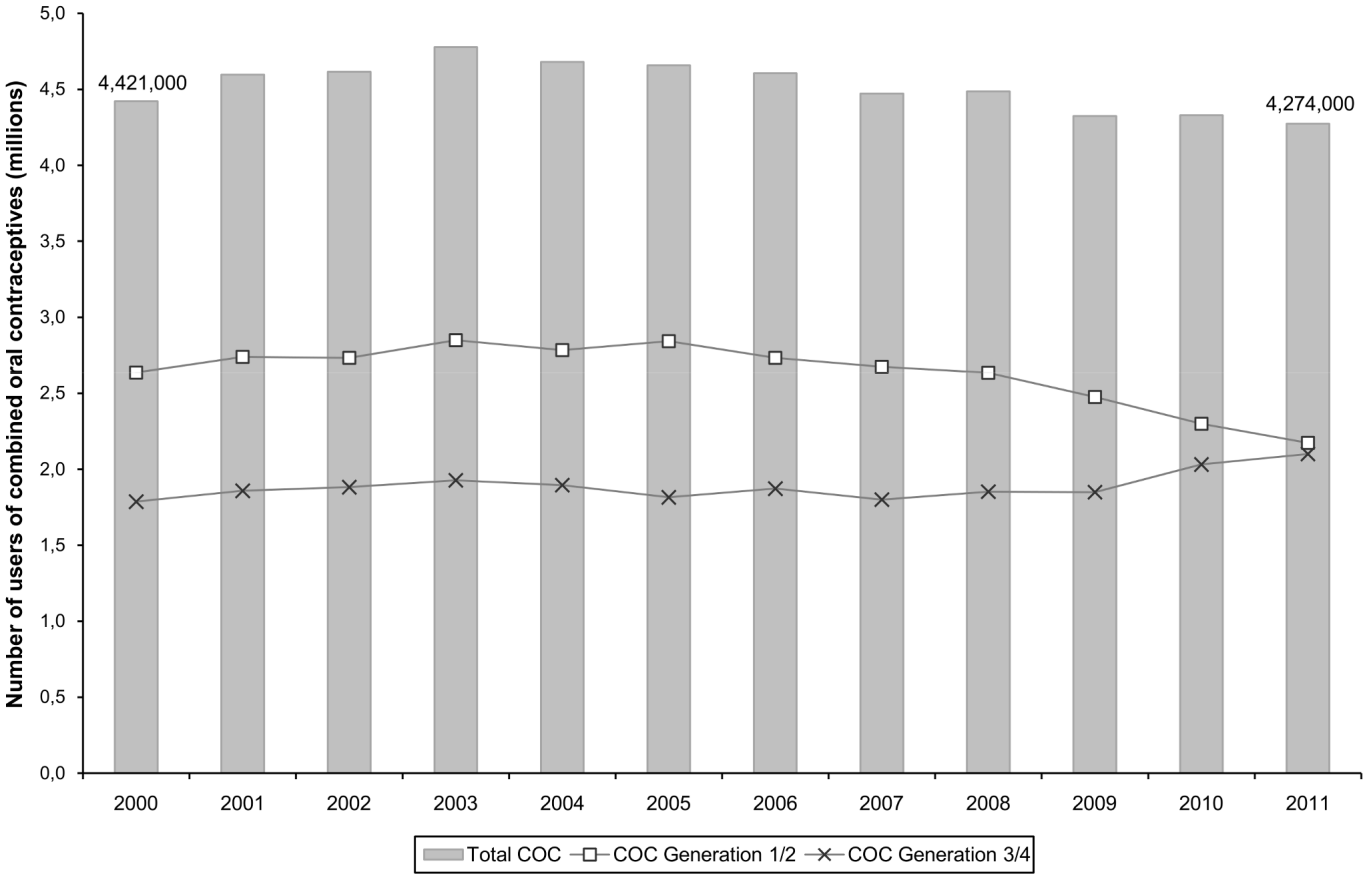
Exposure of combined oral contraceptives, overall and by generation, in France from 2000 to 2011.

In France, from 2000 to 2011, the mean annual number of women exposed every day to a combined oral contraceptive was estimated to 4 519 838 (i.e. an average of 30.3% of the French population of women of childbearing age). Nevertheless, changes in contraceptive utilisation were found over time. Since 2003, the number of women daily exposed has decreased to reach 4 274 000 users in 2011. Furthermore, if approximately 60% of women were users of a first- or second-generation until 2008, they were only 50% in 2011. An increase in third-/fourth-generation users began in 2009 to the detriment of third- or fourth-generation (figure 1).

Data from national surveys indicated that the reduction in the use of contraception was not uniform within five year age groups (tables 3 and 4). The most significant reduction was observed in women less than 35 years old. In women aged over 40, the reduction was close to null. Evolution of women exposure to combined oral contraceptives within five years age groups, from 2000 to 2011, is detailed in appendix S2.

**Table 3:** Exposure of combined oral contraceptives, overall and by generation, in France - Results of Cocon survey, 2000.

Age group (years)	COC*(%)	G1/G2**(%)	G3/G4**(%)
15–17 Y	Not available		
18–19 Y	49,0	73,4	26,6
20–24 Y	55,8	60,4	39,6
25–29 Y	46,9	61,7	38,3
30–34 Y	38,5	61,2	38,8
35–39 Y	27,9	64,5	35,5
40–45 Y	23,4	57,8	42,2
46–49 Y	Not available		

*Percentage of use within the general population.

**Percentage of use within the users of combined oral contraceptives (COC).

**Table 4:** Exposure of combined oral contraceptives, overall and by generation, in France - Results of Fecond survey, 2010.

Age group (years)	COC*(%)	G1/G2**(%)	G3/G4**(%)
15–17 Y	13,0	41,9	58,1
18–19 Y	40,0	53,7	46,3
20–24 Y	47,8	58,0	42,0
25–29 Y	38,6	57,0	43,0
30–34 Y	28,6	55,6	44,4
35–39 Y	23,3	56,2	43,8
40–44 Y	22,1	62,0	38,0
45–49 Y	11,0	63,7	36,3

*Percentage of use within the general population.

**Percentage of use within the users of combined oral contraceptives (COC).

Over the study period, the mean annual number of venous thromboembolic events was estimated to 3891 in women exposed to combined oral contraceptives: 1557 occurred in women exposed to first- and second-generation and 2334 in women exposed to third- and fourth-generation.

**Figure 2:**
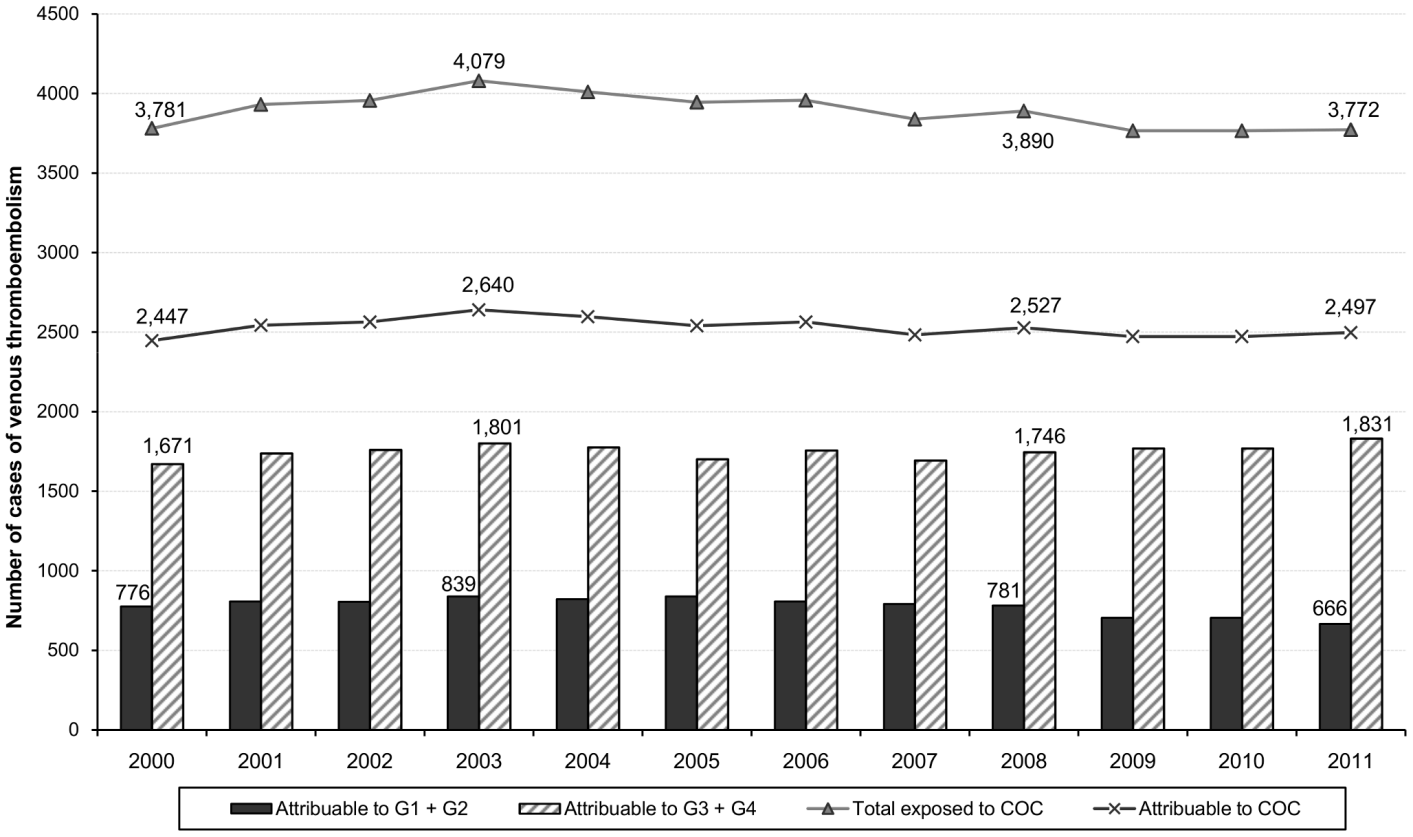
Number of cases of venous thromboembolism annually attributable to combined oral contraceptives. The figure shows the number of cases attributable to combined oral contraceptives overall and by generation in France from 2000 to 2011.

Overall, the mean number of venous thromboembolism attributable to combined oral contraceptives was estimated to 2529 (778 and 1751 cases attributable to first-/second-generation and third-/fourth-generation respectively), corresponding to 843 estimated cases of pulmonary embolism over the study period (figure 2).

**Figure 3:**
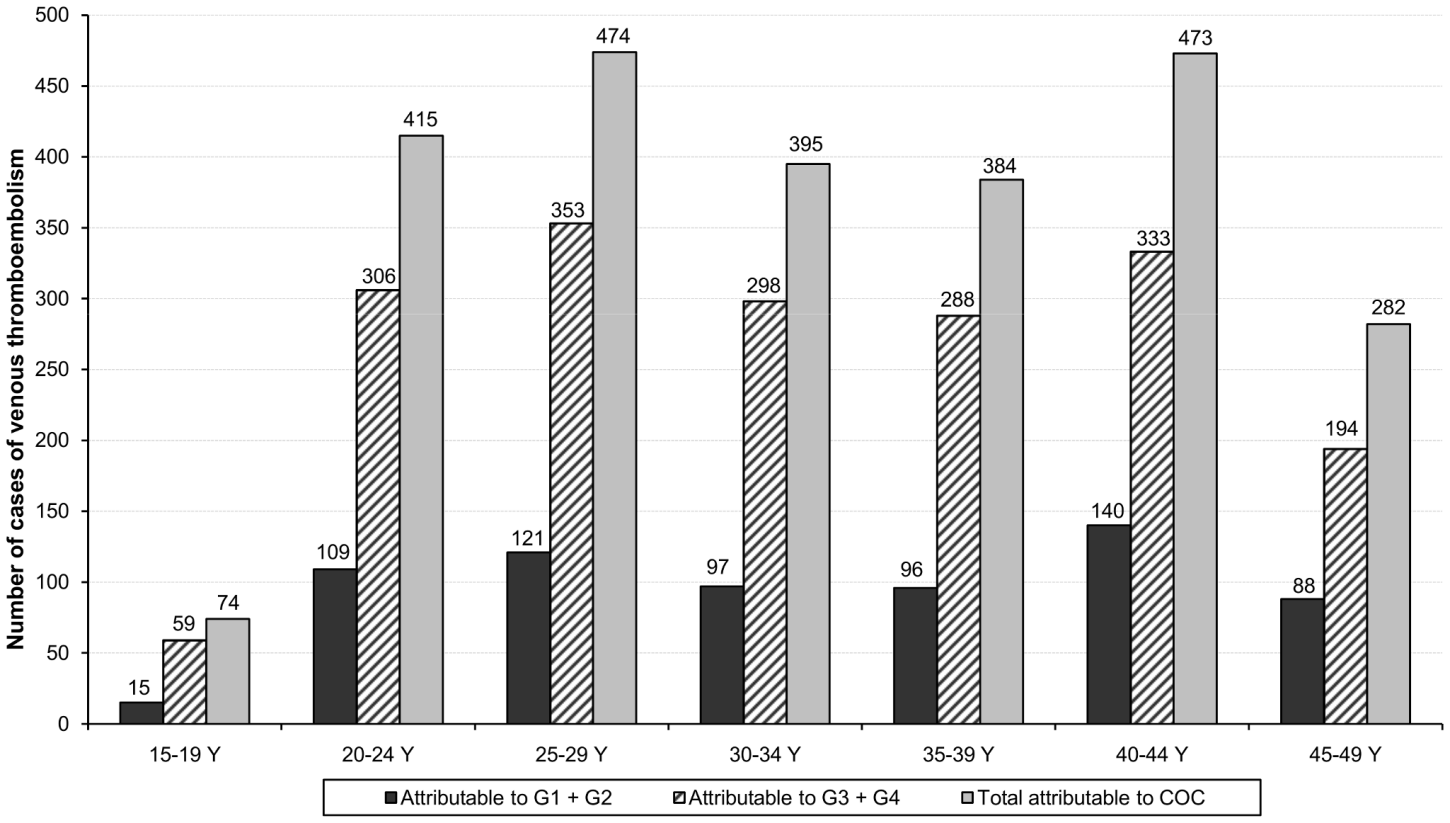
Number of cases of venous thromboembolism attributable to combined oral contraceptives in 2011. The figure shows the number of cases attributable to combined oral contraceptives broken into age group, overall and by generation.

The distribution of estimated venous thromboembolic events attributable to combined oral contraceptives according to age, in 2011, is represented in figure 3. While the highest utilisation rate was observed in women aged 20 to 24 years, with half of women exposed, maximums of cases were observed in women aged 25 to 29 and 40 to 44 years, with 474 and 473 cases respectively. Overall, 31% of users over 35 years contributed to 46% of cases attributable to combined oral contraceptives.

**Figure 4:**
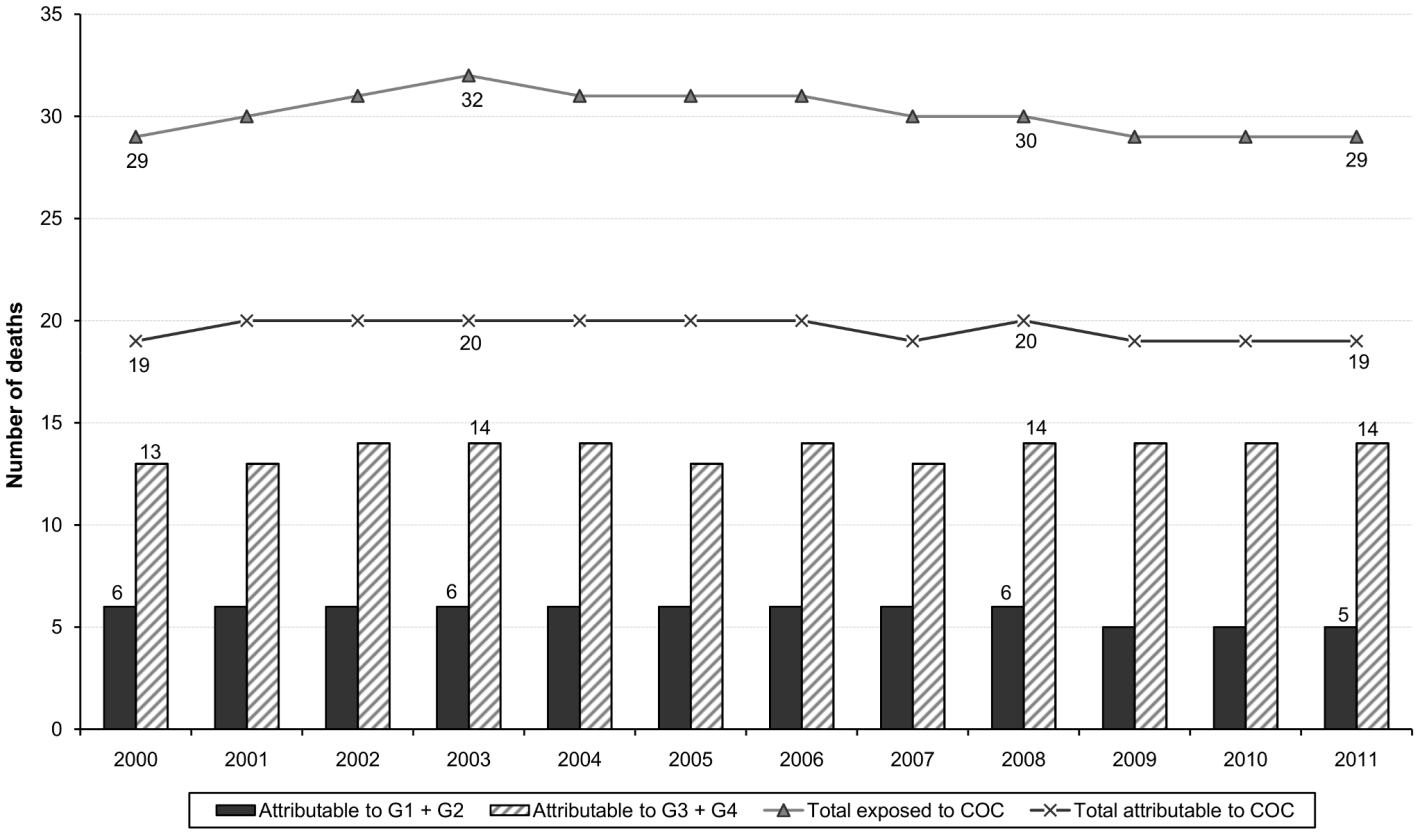
Number of deaths annually attributable to the use of combined oral contraceptives. The figure shows the number of deaths attributable to combined oral contraceptives overall and by generation in France from 2000 to 2011.

The annual median number of premature deaths due to pulmonary embolism attributable to combined oral contraceptives was estimated to 20 (6 and 14 deaths attributable to first-/second-generation and third-/fourth-generation respectively), including eight to nine in-hospital immediate deaths (figure 4).

The mean annual number of venous thromboembolism attributable to third- and fourth-generation contraceptives, as compared to first- and second-generation contraceptives, was estimated to 1167, corresponding to 394 cases of pulmonary embolism. The median number of premature deaths attributable to third- and fourth-generation contraceptives was estimated to nine, including three in-hospital deaths.

Sensitivity analyses combining all extreme hypotheses showed that, in 2011, the estimated number of venous thromboembolism attributable to combined oral contraceptives (compared to non use) ranged from 1762 to 3356 (i.e. 11 to 31 premature deaths). The estimated number of venous thromboembolism attributable to third- and fourth-generation contraceptives (compared to first- and second-generation) ranged from 887 to 1607, i.e. five to fifteen premature deaths. Detailed analyses are provided in appendix S3.

## Discussion

In France, with more than 60 billions blister packs sold each year and more than four millions women exposed each day, combined oral contraceptives are the most widely used option of contraception. Each year, we estimated that 2529 venous thromboembolic events, of which 843 cases of pulmonary embolism and 20 premature deaths were attributable to their use. As compared to first- and second-generation contraceptives, we estimated that exposure to third- and fourth-generation contraceptives led to an annual excess of 1167 venous thromboembolic events and nine premature deaths.

Sales data claimed to the ANSM permitted to determine the mean number of women daily exposed to combined oral contraceptives over the study period, overall and by generation. In France, this number has decreased since 2003 and an increase in users of third- and fourth-generation began in 2009 (year from which third-generation started to be reimbursed) to reach half of combined oral contraceptives users in 2011. As a considerable part of third-generation products are not reimbursed, as well as all fourth-generation products, standard information usually recorded at the time of dispensation are not registered in existing administrative health care databases, such as age of patients. Therefore, we used data from two cross-sectional French surveys conducted in 2000 and 2010 to model the evolution of the combined oral contraceptives utilisation, by generation and by five year age group, and to estimate the annual population of exposed French women. All these separate resources are potential sources of bias. Nevertheless, in our study, sales data and data from surveys were concordant. Therefore, we consider that biases are likely weak.

Absolute risks of a first-lifetime episode of venous thromboembolism retained for our estimations derived from a large Danish study including all Danish women aged 15 to 49 years with neither current or completed thrombotic disease, nor current pregnancy [10]. In this study, incidence rates were estimated in the population of non-users of all types of hormonal contraception, i.e. never users plus former users. We assumed that our study population of non-pregnant French women exposed to combined oral contraceptives was comparable to this population in terms of absence of thrombotic disease, known risk factor or triggering risk of venous thromboembolism (i.e. eligibility criteria to use combined oral contraceptives). Moreover, the absence of evidence to support any European geographic variation of thrombotic disease may allow us to transpose the Danish incidence rates to the French population. However, it should be underlined that users of non-oral combined contraceptives, i.e. vaginal rings and patches, were not excluded from the Danish study and were allocated to the non-exposed group. Later results from the same cohort have shown an increased risk of venous thromboembolism associated to these non-oral products [28]. Nevertheless, given the limited number of women exposed to these products, their non exclusion should have an insignificant impact on estimated absolute risks. The Danish cohort [9], [10], [28], with a power ten times greater than former studies conducted in general population [20], [21], studied women by five year age group. Particularly, its results were in line with those of the only available French study, an older study including a low number of women of childbearing age split into only two age groups: 20–39 and 40–59 years of age [19].

Due to regulation reasons, hospitalisation data before 2007 are not available. Nevertheless, we have assumed that a trend towards either a decrease or an increase of in-hospital lethality rate, if exists, could only be insignificant. Firstly, the study period was conducted over a short period (i.e. 12 years). Secondly, the annual immediate in-hospital lethality rate in non-pregnant women aged 15 to 49 years without history of malignancy was stable between 2007 and 2011. Finally, in France, the pulmonary embolism mortality in general population is stable since 1990 [29].

We compared our estimations obtained from data of the Danish study to the number of hospitalisations for pulmonary embolism reported in the French nationwide hospital medical information database, which collect almost all hospitalisations. After exclusion of hospitalisations occurring in pregnant women or in women with malignancy, the mean annual number of hospitalisations for pulmonary embolism in women aged 15 to 49 years between 2007 and 2011 was 2504 (1040 and 1464 in women aged 15–34 and 35–49 years of age respectively). Over the same period, the number of pulmonary embolism estimated by applying Danish data to the French population of women without current pregnancy or malignancy corresponds to 95% of reported hospitalised pulmonary embolism, i.e. 2382 (968 and 1414 in women aged 15–34 and 35–49 years of age respectively). It should be noted that drug exposure is not collected in the French nationwide hospital medical information database. Thus, although reported cases of pulmonary embolism cannot be related to exposure to combined oral contraceptives, these results highlight the relevancy of the use of Danish absolute risks of venous thromboembolism.

Regarding combined oral contraceptives-associated relative risks of venous thromboembolism, literature data show that certain confounding factors, such as smoking and obesity, are often not considered. However, smoking is not a known major risk factor for venous thromboembolism and analyses performed in other studies with adjustment for body mass index did not modify their results [9], [23], [30]. This former result was also observed in the French survey Cocon. Our estimations of the number of venous thromboembolic events could have been overestimated. Indeed, given the lack of studies aiming at assessing the risk of venous thromboembolism in users of fourth-generation products containing chlormadinone, dienogest or nomegestrol, we assumed that these products carried a risk similar to that of the third-generation products [10]–[12]. However, population of exposed women to these products is very low (7.9% among users of fourth-generation combined oral contraceptives over the last five years), so that consequences of such a conservative hypothesis should be minor. The current study has investigated the premature mortality due to pulmonary embolism defined as mortality within five years due to recurrent pulmonary embolism, including immediate in-hospital lethality. The immediate out-hospital lethality due to pulmonary embolism is unknown and could not have been taken into account. Nevertheless, such lethality could be assumed to be very low in young women. Our work is limited to the estimation of the number of venous thromboembolic events attributable to combined oral contraceptives; the risk of arterial thromboembolism (stroke and myocardial infarction) was not assessed. It should also be noted that we limited our study to events associated to combined oral contraceptives; hence we did not consider events associated to the combination ethinylestradiol/cyproterone acetate, which is only indicated in the treatment of acne in France. The increased risk carried by these products is nevertheless similar to the one of third- and fourth-generation containing drospirenone combined oral contraceptive [10].

Finally, our results show that, due to the considerable number of women exposed to third- and fourth-generation products, 1167 venous thromboembolic events on average each year might be prevented whether all women used first- or second-generation products. This excess of venous thromboembolism corresponds to nine deaths in the upcoming five years, of which three immediate in-hospital deaths.

Our results cannot be generalized to other European countries since they are only based on French data. In particular, data of exposure to combined oral contraceptives by five year age groups and by generation are not available at European level. Nevertheless, we performed a crude analysis in five European countries (France, Germany, Italy, Spain, and United Kingdom). We applied both the overall incidence rate of venous thromboembolism from the Danish study [10] and the retained relative risks in our study to the number of users estimated from sales data at national level [31]. Overall, in 2011, in these countries, which account for approximately 63% of European Union inhabitants, exposure to third- and fourth-generation contraceptives led to an excess of 7764 venous thromboembolic events and 60 premature deaths due to pulmonary embolism, as compared to the use of first- and second-generation contraceptives. Reduction in the use of third- and fourth-generation pills is therefore essential. All combined oral contraceptives are equally effective in preventing pregnancy. Their side effects and benefits (notably in terms of acne) would be similar according to an editorial, which recently recalled that the only acceptable strategy is to use the safest combined oral contraceptive with regard to venous thrombosis, i.e. the one that contains the lowest tolerable dose of ethinylestradiol together with the second generation progestogen [32].

More generally, for each woman, it is necessary to examine which is the most appropriate contraceptive method in terms of both acceptability and risks. Although they have been widely prescribed over the last four decades, combined oral contraceptives should not be the only suggested contraception option, even for first- and second-generation combined oral contraceptives. For all women opting for such products, the following risk factors for venous thromboembolism should be investigated before any prescription is made: obesity, increasing age, positive family history, prolonged immobilisation, major surgery, any surgery to the legs or pelvis, neurosurgery, or major trauma, and medical conditions associated with venous thromboembolism such as cancer, systemic lupus erythematosus, haemolytic uraemic syndrome, chronic inflammatory bowel disease and sickle cell disease [33]. Moreover, physicians and women should be aware of any risks and warning signs which could lead to consult without delay for an early medical management.

## Conclusion

Each year in France, more than four millions women are daily exposed to combined oral contraceptives. We estimated that, annually on average, 2529 venous thromboembolic events were attributable to their use. The mean annual number of venous thromboembolism attributable to third- and fourth-generation contraceptives was estimated to 1167, corresponding to nine premature deaths, including three immediate in-hospital deaths. Consequently, corrective actions should be considered in order to limit exposure to third- and fourth- generation contraceptives, and thus optimise the benefit-risk ratio of combined oral contraception.

## Supporting Information

Appendix S1 **Detailed methodology for the estimation of the number of women exposed to combined oral contraceptives by age and by generation from 2000 to 2011**
(DOC)

Appendix S2 **Evolution of exposure to combined oral contraceptives by age, in France in 2000, 2003 and 2011**
(DOC)

Appendix S3 **Sensitivity analyses**
(DOC)
