# Supplementary material for: Estimate of Venous Thromboembolism and Related-Deaths Attributable to the Use of Combined Oral Contraceptives in France
Source: PLoS One. 2014 Apr 21;9(4):e93792. doi: 10.1371/journal.pone.0093792 (PMC3994005; doi:10.1371/journal.pone.0093792)
Supplement: Appendix S1 — Detailed methodology for the estimation of the number of women exposed to combined oral contraceptives by age and by generation from 2000 to 2011 (DOC) [file pone.0093792.s001.doc]

**Appendix S1**

**Detailed methodology for the estimation of the number of women exposed to combined oral contraceptives by age and by generation from 2000 to 2011**

Required data to estimate the number of women exposed to combined oral contraceptives, by age, for a given year were the followings:

- Estimation of the French population by year and by five year age group (15-49 years)

Sources: Data on the French population census from the French National Institute of Statistics (Insee)

- Estimation of the number of women annually exposed to a combined oral contraceptive, overall, by generation and by age

Sources: sales of combined oral contraceptives annually claimed to the ANSM and data from French cross-sectional surveys investigating contraception behaviours performed in 2000 and 2010

1. **Data presentation**
   1. Data from French national census (Insee)

We used data from the source ‘Estimations de population’ for continental France and overseas departments and territories (apart from Mayotte) that provide French population broken down into sex and five year age group on first January of calendar year [1].

Used data are those of women aged from 15 to 49 years of age from 2000 to 2011. Women of the 15-19 years of age group were distributed into two distinct groups to take into account the heterogeneity of contraception behaviours within this five year age group on the assumption that each one year age group is equally distributed (15-17 (three years) and 18-19 years old (two years)).

- 1. Sales data claimed to the ANSM

Data on sales of combined oral contraceptives annually claimed to the ANSM have been used to estimate the mean number of women daily exposed to a combined oral contraceptive over the period 2000-2011, overall and by generation (first-/second-generation versus third-/fourth-generation) [2,3].

*Number of women exposed to a combined oral contraceptive*

The mean number of daily exposed women was estimated by dividing the number of blister packs claimed each year by 13, i.e. the maximum number of blister packs used per woman and per year. The number of daily exposed women increased until 2003, then has decreased since 2004 (figure S1).

*Distribution of women exposed to a combined oral contraceptive by generation*

From 2000 to 2008, about 40% of women exposed to a combined oral contraceptive used a third- or fourth-generation product and 60% a first- or second-generation product. This distribution was constant over the eight year period. Since 2009, users of third- and fourth-generation products have increased to the detriment of first- or second-generation products to reach half of combined oral contraceptives users in 2011 (figure S2).

- 1. French survey data on contraception behaviours

In France, several surveys investigating contraception behaviours have been set up since 1978; all were conducted in representative samples of women of childbearing age.

Data used in the scope of this study derived from the two more recent surveys:

- The Cocon survey, performed in 2863 women aged 18-44 years in 2000 [4],
- The Fecond survey, conducted over the phone in 5275 women aged 15-49 years in 2010 [5].

Results of both French surveys regarding combined oral contraception use, Cocon and Fecond, are summarized in tables 1 and 2.

*Use of combined oral contraceptives, overall*

Comparison between data from both French cross-sectional surveys indicates a decrease of the use of combined oral contraceptives between 2000 and 2010 (Tables S1 and S2, figure S3). This reduction was not uniform within five year age groups. The most significant reduction was observed in women less than 35 years old. In women aged 40 years and more, the diminution was close to null.

*Use of combined oral contraceptives, by generation*

In combined oral contraceptives users aged less than 40 years, the percentage of users of third- and fourth-generation products (on a base 100) increased (+74% in users aged 18-19 years and from 6 to 23% in users aged 20-39 years compared to data from 2000 (Tables S1 and S2, figure S4)). In 2010, the majority of users aged 15-17 years used third- and fourth-generation products.

1. **Estimation of combined oral contraceptives exposure by age and generation from 2000 to 2011**

Data from ANSM and national surveys were studied in order to draw the most relevant working hypotheses to define sub-period study. Indeed, insofar as no data were available by age group apart from 2000 and 2010, there was a need to determine the most appropriate period for which data from one or other of the two surveys could apply, notably according to the spreading of first-/second-generation and third-/fourth-generation within each age group.

Considering that:

- The mean number of women exposed to a combined oral contraceptive was increasing from 2000 to 2003 and was decreasing from 2004 to 2011 (sales data claimed to ANSM).
- A decrease in the use of combined oral contraceptives between 2000 and 2010 was observed from French national surveys data; the reduction was not homogeneous within five year age groups.
- An increase in the percentage of users of third- and fourth-generation combined oral contraceptives, stable until then, began in 2009. This modification of the spreading of generations of pills (first- and second- versus third- and fourth- generation) was homogeneous within five year age groups.

Three distinct periods were thus defined: 2000-2003, 2004-2008 and 2009-2011. Following hypotheses have been advanced for the estimation of the number of women exposed to combined oral contraceptives by age and by generation:

*First and second periods: 2000-2003 and 2004-2008*

For the period 2000-2008, the number of women annually exposed to a combined oral contraceptive, by age and by generation, was estimated within each age groups by applying utilisation rates derived from the Cocon survey (first-/second-generation and third-/fourth-generation) to the number of French women of the considered age group (from data on French population census). The spreading of first-/second-generation and third-/fourth-generation was assumed to be stable over the nine year period. For the extreme age group not included in the Cocon survey (i.e. 15-17 and 46-49 years), following hypotheses were advanced to estimate utilisation rates of combined oral contraceptives within each age group:

- The mean number of users aged 15 to 17 years remained stable over the study period 2000-2011, close to 13.0%. This assumption is supported by the stability of women age at sexual initiation over the period, as well as the significant use of non oral contraceptives (mainly condoms) within this age group. Utilisation rate regarding the spreading of first-/second-generation and third-/fourth-generation were those of the adjacent age group (i.e. 18-19 years) from Cocon survey.
- For women aged 46 to 49 years, two estimates were available: the first derived from the national Ined/Inserm survey conducted in 1974 in 2944 women aged 20 to 49 years, ^6^ and the second from the Fecond survey. Utilisation rates were 14.5% in 174 and 19.0% 2010. Linear interpolation yielded an overall utilisation rate about 16.2%. Utilisation rate regarding the spreading of first-/second-generation and third-/fourth-generation were those of the adjacent age group (i.e. 40-45 years) from Cocon survey.

Tables 4 summarises data from national surveys (raw data or estimates) used to estimate the number of women exposed to combined oral contraceptives by age and generation from 2000 to 2008.

For the first period (2000-2003), the estimation was adjusted with the estimation of the mean number of exposed women from sales data claimed to ANSM, by applying the same coefficient within each age group for the given year. The advanced hypothesis is that the observed increased is uniform within each age group.

During the second period (2004-2008), the number of women exposed to combined oral contraceptives decreased independently of age. Slopes relating to each age group were calculated from both national surveys data (Cocon and Fecond) on the assumption of a linear reduction from 2003 to 2010 (table 3), and applied to the estimation the number of women exposed within each age group from 2004 to 2008. The estimation was then adjusted with sales data claimed to ANSM for the given year (as described above).

*Last period: 2009-2011*

For the third period (2009-2011), utilisation rates of the Fecond survey were used to estimate the number of women annually exposed to a combined oral contraceptive, by generation, within each age groups (i.e. 15-17, 18-19, 20-24, 25-29, 30-34, 35-39, 40-44, 45-49 years). The estimation was then adjusted with sales data claimed to ANSM for the given year (as described above). The spreading of first-/second-generation and third-/fourth-generation is assumed to be stable over the three year period.

**REFERENCES**

**TABLES**

**Table S1 - Appendix S1: Exposure of combined oral contraceptives, overall and by generation, in France - Results of Cocon survey, 2000**

| **Age group (years)** | **COC*(%)** | **G1/G2**(%)** | **G3/G4**(%)** |
| --- | --- | --- | --- |
| 15-17 Y | Not available | | |
| 18-19 Y | 49,0 | 73,4 | 26,6 |
| 20-24 Y | 55,8 | 60,4 | 39,6 |
| 25-29 Y | 46,9 | 61,7 | 38,3 |
| 30-34 Y | 38,5 | 61,2 | 38,8 |
| 35-39 Y | 27,9 | 64,5 | 35,5 |
| 40-45 Y | 23,4 | 57,8 | 42,2 |
| 46-49 Y | Not available | | |

* Percentage of use within the general population

** Percentage of use within the users of combined oral contraceptives (COC)

**Table S2 - Appendix S1: Exposure of combined oral contraceptives, overall and by generation, in France - Results of Fecond survey, 2010**

| **Age group (years)** | **COC*(%)** | **G1/G2**(%)** | **G3/G4**(%)** |
| --- | --- | --- | --- |
| 15-17 Y | 13,0 | 41,9 | 58,1 |
| 18-19 Y | 40,0 | 53,7 | 46,3 |
| 20-24 Y | 47,8 | 58,0 | 42,0 |
| 25-29 Y | 38,6 | 57,0 | 43,0 |
| 30-34 Y | 28,6 | 55,6 | 44,4 |
| 35-39 Y | 23,3 | 56,2 | 43,8 |
| 40-44 Y | 22,1 | 62,0 | 38,0 |
| 45-49 Y | 11,0 | 63,7 | 36,3 |

* Percentage of use within the general population

** Percentage of use within the users of combined oral contraceptives (COC)

**Table S3 - Appendix S1: Slopes relating to utilisation rate of combined oral contraceptives within age group from 2003 to 2010**

|  | **18-19 Y** | **20-24 Y** | **25-29 Y** | **30-34 Y** | **35-39 Y** | **40-45 Y** |
| --- | --- | --- | --- | --- | --- | --- |
| **Slope** | - 1,0 % /year | - 0,9 % /year | - 1,0 % /year | - 1,2 % /year | - 0,5 % /year | - 0,01% /year |

**Table S4 - Appendix S1: Utilisation rates** of combined oral contraceptives by age in 2000 (Ined/Inserm surveys)

|  | **15-17 Y** | **18-19 Y** | **20-24 Y** | **25-29 Y** | **30-34 Y** | **35-39 Y** | **40-45 Y** | **46-49 Y** |
| --- | --- | --- | --- | --- | --- | --- | --- | --- |
| % COC | 13,0* | 49,0 | 55,8 | 46,9 | 38,5 | 27,9 | 23,4 | 13,6* |
| % G1 + G2 | 9,5* | 35,9 | 33,7 | 29,0 | 23,6 | 18,0 | 13,5 | 7,9* |
| % G3 + G4 | 3,5* | 13,0 | 22,1 | 18,0 | 14,9 | 9,9 | 9,9 | 5,7* |

* Estimates

**FIGURES**

Figure S1 - Appendix S1: Mean number of daily exposed women to a combined oral contraceptive in France from 2000 to 2011

Figure S2 - Appendix S1: Distribution of women exposed to a combined oral contraceptive by generation in France from 2000 to 2011

Figure S3 - Appendix S1: Utilisation rate of combined oral contraceptives by age in 2000 and 2010 (Ined/Inserm surveys)

Figure S4 - Appendix S1: Utilisation rate of third- and fourth- generation combined oral contraceptives by age in 2000 and 2010 (Ined / Inserm surveys)
