# Supplementary material for: Estimate of Venous Thromboembolism and Related-Deaths Attributable to the Use of Combined Oral Contraceptives in France
Source: PLoS One. 2014 Apr 21;9(4):e93792. doi: 10.1371/journal.pone.0093792 (PMC3994005; doi:10.1371/journal.pone.0093792)
Supplement: Appendix S2 — Evolution of exposure to combined oral contraceptives by age, in France in 2000, 2003 and 2011 (DOC) [file pone.0093792.s002.doc]

**Appendix S2**

**Evolution of exposure to combined oral contraceptives by age, in France in 2000, 2003 and 2011**

Figure S1 - Appendix S2: Evolution of overall exposure to combined oral contraceptives by age, in France in 2000, 2003 and 2011

Figure S2 - Appendix S2: Evolution of exposure to combined oral contraceptives by age and by generation (first- and second-generation versus third- and fourth-generation) in 2000, 2003 and 2011
