# Supplementary material for: Estimate of Venous Thromboembolism and Related-Deaths Attributable to the Use of Combined Oral Contraceptives in France
Source: PLoS One. 2014 Apr 21;9(4):e93792. doi: 10.1371/journal.pone.0093792 (PMC3994005; doi:10.1371/journal.pone.0093792)
Supplement: Appendix S3 — Sensitivity analyses (DOC) [file pone.0093792.s003.doc]

**Appendix S3**

### Sensitivity analyses

1. **Analysis no.1 : Variation of absolute incidence rates of venous thromboembolism**

A first sensitivity analysis was conducted using lower and upper limits of the 95% confidence intervals (CI) of incidence rates (IR) of venous thromboembolism (VTE) from the Danish study [1].

In 2011, the estimated number of venous thromboembolic events attributable to the use of combined oral contraceptives (compared to non use) ranged from 2137 to 2855 (i.e. from 712 to 952 cases of pulmonary embolism and from 17 to 22 deaths in the five upcoming years). The estimated number of venous thromboembolic events attributable to third- and fourth-generation products (compared to the use of first- and second-generation products) ranged from 1043 to 1397 (tables S1-4).

1. **Analysis no.2 : Variation of combined oral contraceptives-associated relative risk of venous thromboembolism**

A second analysis was performed by varying combined oral contraceptives-associated relative risk of venous thromboembolism associated to the use of first- and second-generation products (versus non use) and third- and fourth-generation products (versus non use and first- and second-generation products) with the extreme values the most frequently founded in the literature [1–10].

In 2011, the estimated number of venous thromboembolic events attributable to the use of combined oral contraceptives (compared to non use) ranged from 2058 to 2934 (i.e. from 686 to 978 cases of pulmonary embolism and from 16 to 23 deaths in the five upcoming years). The estimated number of venous thromboembolic events attributable to third- and fourth-generation products (compared to the use of first- and second-generation products) ranged from 1037 to 1403 (tables S5-8).

1. **Analysis no.3 : Variation of in-hospital lethality rate**

A third sensitivity analysis was conducted using lower and upper limits of the 95% confidence intervals (CI) of in-hospital lethality due to pulmonary embolism rate.

In 2011, the estimated number of in-hospital deaths due to pulmonary embolism attributable to combined oral contraceptives (compared to non use) ranged from 6 to 10 (table S9).

1. **Analysis no.4 : Variation of premature mortality rate**

A fourth sensitivity analysis was conducted using lower and upper limits of the 95% confidence intervals (CI) of premature mortality due to pulmonary embolism rate.

In 2011, the estimated number of premature deaths due to pulmonary embolism in the five upcoming years attributable to combined oral contraceptives (compared to non use) ranged from 15 to 23 (table S10).

1. **Analysis no.5 : Combination of extreme hypotheses**

A last sensitivity analysis was performed combining all previous sensitivity analysis, i.e. using lower and upper limits of the 95% CI of IR of VTE from the Danish study [1] and mortality due to pulmonary embolism rates (in-hospital lethality rate and premature mortality rate), and varying combined oral contraceptives-associated relative risk of VTE associated to the use of first- and second-generation products (versus non use) and third- and fourth-generation products (versus non use and first- and second-generation products).

In 2011, the estimated number of venous thromboembolism attributable to combined oral contraceptives (compared to non use) ranged from 1762 to 3356 (i.e. from 587 to 1119 cases of pulmonary embolism and from 11 to 31 premature deaths in the five upcoming years). The estimated number of venous thromboembolism attributable to third- and fourth-generation contraceptives (compared to first- and second-generation) ranged from 887 to 1607, i.e. five to fifteen premature deaths (tables S11-14).

**REFERENCES**

1. Lidegaard Ø, Nielsen LH, Skovlund CW, Skjeldestad FE, Løkkegaard E (2011) Risk of venous thromboembolism from use of oral contraceptives containing different progestogens and oestrogen doses: Danish cohort study, 2001-9. BMJ 343: d6423.

2. World Health Organization Collaborative Study of Cardiovascular Disease and Steroid Hormone Contraception (1995) Effect of different progestagens in low oestrogen oral contraceptives on venous thromboembolic disease. Lancet 346: 1582–1588.

3. Jick H, Jick SS, Gurewich V, Myers MW, Vasilakis C (1995) Risk of idiopathic cardiovascular death and nonfatal venous thromboembolism in women using oral contraceptives with differing progestagen components. Lancet 346: 1589–1593.

4. Bloemenkamp KW, Rosendaal FR, Helmerhorst FM, Büller HR, Vandenbroucke JP (1995) Enhancement by factor V Leiden mutation of risk of deep-vein thrombosis associated with oral contraceptives containing a third-generation progestagen. Lancet 346: 1593–1596.

5. Spitzer WO, Lewis MA, Heinemann LA, Thorogood M, MacRae KD (1996) Third generation oral contraceptives and risk of venous thromboembolic disorders: an international case-control study. Transnational Research Group on Oral Contraceptives and the Health of Young Women. BMJ 312: 83–88.

6. Jordan WM, Anand JK (1961) Pulmonary embolism. The Lancet 278: 1146–1147. doi:10.1016/S0140-6736(61)91061-3.

7. Boyce J, Fawcett JW, Noall EW (1963) Coronary thrombosis and Conovide. Lancet 1: 111.

8. Inman WH, Vessey MP, Westerholm B, Engelund A (1970) Thromboembolic disease and the steroidal content of oral contraceptives. A report to the Committee on Safety of Drugs. Br Med J 2: 203–209.

9. Parkin L, Sharples K, Hernandez RK, Jick SS (2011) Risk of venous thromboembolism in users of oral contraceptives containing drospirenone or levonorgestrel: nested case-control study based on UK General Practice Research Database. BMJ 342: d2139.

10. Jick SS, Hernandez RK (2011) Risk of non-fatal venous thromboembolism in women using oral contraceptives containing drospirenone compared with women using oral contraceptives containing levonorgestrel: case-control study using United States claims data. BMJ 342: d2151.

**TABLES**

Table S1 - Appendix S3: Number of venous thromboembolic events (VTE) and related deaths attributable to the use of combined oral contraceptives (overall), results from sensitivity analysis on variation of absolute incidence rates of VTE

| **Estimated number of attributable cases *** | **Hypothesis: lower limits of the 95% CI of IR from Lidegaard **** | ***Working hypothesis: IR from Lidegaard*** | **Hypothesis: upper limits of the 95% CI of IR from Lidegaard **** |
| --- | --- | --- | --- |
| VTE | 2137 | 2497 | 2855 |
| In-hospital deaths | 7 | 8 | 9 |
| Premature deaths | 17 | 19 | 22 |

* Number of cases estimated within each five year age group, pooled results

** Data from table 1 of the core text

Table S2 - Appendix S3: Number of venous thromboembolic events (VTE) and related deaths attributable to the use of G1 + G2 combined oral contraceptives, results from sensitivity analysis on variation of absolute incidence rates of VTE

| **Estimated number of attributable cases *** | **Hypothesis: lower limits of the 95% CI of IR from Lidegaard **** | ***Working hypothesis: IR from Lidegaard*** | **Hypothesis: upper limits of the 95% CI of IR from Lidegaard **** |
| --- | --- | --- | --- |
| VTE | 572 | 666 | 759 |
| In-hospital deaths | 2 | 2 | 2 |
| Premature deaths | 4 | 5 | 6 |

* Number of cases estimated within each five year age group, pooled results

** Data from table 1 of the core text

Table S3 - Appendix S3: Number of venous thromboembolic events (VTE) and related deaths attributable to the use of G3 + G4 combined oral contraceptives, results from sensitivity analysis on variation of absolute incidence rates of VTE

| **Estimated number of attributable cases *** | **Hypothesis: lower limits of the 95% CI of IR from Lidegaard **** | ***Working hypothesis: IR from Lidegaard*** | **Hypothesis: upper limits of the 95% CI of IR from Lidegaard **** |
| --- | --- | --- | --- |
| VTE | 1565 | 1831 | 2096 |
| In-hospital deaths | 5 | 6 | 6 |
| Premature deaths | 12 | 14 | 16 |

* Number of cases estimated within each five year age group, pooled results

** Data from table 1 of the core text

Table S4 - Appendix S3: Number of venous thromboembolic events (VTE) and related deaths attributable to the use of G3 + G4 combined oral contraceptives (compared to G1 + G2), results from sensitivity analysis on variation of absolute incidence rates of VTE

| **Estimated number of attributable cases *** | **Hypothesis: lower limits of the 95% CI of IR from Lidegaard **** | ***Working hypothesis: IR from Lidegaard*** | **Hypothesis: upper limits of the 95% CI of IR from Lidegaard **** |
| --- | --- | --- | --- |
| VTE | 1043 | 1220 | 1397 |
| In-hospital deaths | 3 | 3 | 4 |
| Premature deaths | 8 | 9 | 11 |

* Number of cases estimated within each five year age group, pooled results

** Data from table 1 of the core text

Table S5 - Appendix S3: Number of venous thromboembolic events (VTE) and related deaths attributable to the use of combined oral contraceptives (overall), results from sensitivity analysis on variation of combined oral contraceptives-associated relative risk of VTE

| **Estimated number of attributable cases *** | **‘Low risk’ hypothesis** | ***Working hypothesis*** | **‘High risk’ hypothesis** |
| --- | --- | --- | --- |
|  | **RR G1-G2/NE** : 1,8** | **RR G1-G2/NE : 2,0** | **RR G1-G2/NE : 2,2** |
|  | **RR G3-G4/NE : 3,5** | **RR G3-G4/NE : 4,0** | **RR G3-G4/NE : 4,5** |
| VTE | 2058 | 2497 | 2934 |
| In-hospital deaths | 6 | 8 | 9 |
| Premature deaths | 16 | 19 | 23 |

* Number of cases estimated within each five year age group, pooled results

** NE: non exposed women

Table S6 - Appendix S3: Number of venous thromboembolic events (VTE) and related deaths attributable to the use of G1 + G2 combined oral contraceptives, results from sensitivity analysis on variation of combined oral contraceptives-associated relative risk of VTE

| **Estimated number of attributable cases *** | **‘Low risk’ hypothesis** | ***Working hypothesis*** | **‘High risk’ hypothesis** |
| --- | --- | --- | --- |
|  | **RR G1-G2/NE** : 1,8** | **RR G1-G2/NE : 2,0** | **RR G1-G2/NE : 2,2** |
|  | **RR G3-G4/NE : 3,5** | **RR G3-G4/NE : 4,0** | **RR G3-G4/NE : 4,5** |
| VTE | 533 | 666 | 799 |
| In-hospital deaths | 2 | 2 | 3 |
| Premature deaths | 4 | 5 | 6 |

* Number of cases estimated within each five year age group, pooled results

** NE: non exposed women

Table S7 - Appendix S3: Number of venous thromboembolic events (VTE) and related deaths attributable to the use of G3 + G4 combined oral contraceptives, results from sensitivity analysis on variation of combined oral contraceptives-associated relative risk of VTE

| **Estimated number of attributable cases *** | **‘Low risk’ hypothesis** | ***Working hypothesis*** | **‘High risk’ hypothesis** |
| --- | --- | --- | --- |
|  | **RR G1-G2/NE** : 1,8** | **RR G1-G2/NE : 2,0** | **RR G1-G2/NE : 2,2** |
|  | **RR G3-G4/NE : 3,5** | **RR G3-G4/NE : 4,0** | **RR G3-G4/NE : 4,5** |
| VTE | 1525 | 1831 | 2135 |
| In-hospital deaths | 5 | 6 | 7 |
| Premature deaths | 12 | 14 | 17 |

* Number of cases estimated within each five year age group, pooled results

** NE: non exposed women

Table S8 - Appendix S3: Number of venous thromboembolic events (VTE) and related deaths attributable to the use of G3 + G4 combined oral contraceptives (compared to G1 + G2), results from sensitivity analysis on variation of combined oral contraceptives-associated relative risk of VTE

| **Estimated number of attributable cases *** | **‘Low risk’ hypothesis** | ***Working hypothesis*** | **‘High risk’ hypothesis** |
| --- | --- | --- | --- |
|  | **RR G1-G2/NE** : 1,8** | **RR G1-G2/NE : 2,0** | **RR G1-G2/NE : 2,2** |
|  | **RR G3-G4/NE : 3,5** | **RR G3-G4/NE : 4,0** | **RR G3-G4/NE : 4,5** |
| VTE | 1037 | 1220 | 1403 |
| In-hospital deaths | 3 | 3 | 4 |
| Premature deaths | 8 | 9 | 11 |

* Number of cases estimated within each five year age group, pooled results

** NE: non exposed women

Table S9 - Appendix S3: Number of in-hospital deaths due to pulmonary embolism attributable to the use of combined oral contraceptives, results from sensitivity analysis on variation of in-hospital lethality rate

| **Estimated number of attributable in-hospital deaths *** | **‘Low risk’ hypothesis** | ***Working hypothesis*** | **‘High risk’ hypothesis** |
| --- | --- | --- | --- |
|  | **Rate _15-34 Y_: 0,4%** | **Rate _15-34 Y_: 0,6%** | **Rate _15-34 Y_: 0,8%** |
|  | **Rate _35-49 Y_: 1,1%** | **Rate _35-49 Y_: 1,33%** | **Rate _35-49 Y_: 1,6%** |
| Deaths attributable to the use of COC (overall) | 6 | 8 | 10 |
| Deaths attributable to the use of G1 + G2 | 2 | 2 | 3 |
| Deaths attributable to the use of G3 + G4 | 4 | 6 | 7 |
| Deaths attributable to the use of G3 + G4 (compared to G1 + G2) | 3 | 3 | 5 |

* Number of cases estimated within each five year age group, pooled results

Table S10 - Appendix S3: Number of premature deaths due to pulmonary embolism attributable to the use of combined oral contraceptives, results from sensitivity analysis on variation of in-hospital lethality rate

| **Estimated number of premature deaths *** | **‘Low risk’ hypothesis** | ***Working hypothesis*** | **‘High risk’ hypothesis** |
| --- | --- | --- | --- |
|  | **Rate : 1,8%** | **Rate : 2,3%** | **Rate : 2,8%** |
| Deaths attributable to the use of COC (overall) | 15 | 19 | 23 |
| Deaths attributable to the use of G1 + G2 | 4 | 5 | 6 |
| Deaths attributable to the use of G3 + G4 | 11 | 14 | 17 |
| Deaths attributable to the use of G3 + G4 (compared to G1 + G2) | 7 | 9 | 11 |

* Number of cases estimated within each five year age group, pooled results

Table S11 - Appendix S3: Number of venous thromboembolic events (VTE) and related deaths attributable to the use of combined oral contraceptives (overall), results from combined sensitivity analyses

| **Estimated number of attributable cases *** | **‘Low risk’ hypothesis** | ***Working hypothesis*** | **‘High risk’ hypothesis** |
| --- | --- | --- | --- |
|  | **Lower limits of the 95% CI of IR from Lidegaard **** | ***IR from Lidegaard*** | **Upper limits of the 95% CI of IR from Lidegaard **** |
|  | **RR G1-G2/NE** : 1,8** | **RR G1-G2/NE : 2,0** | **RR G1-G2/NE : 2,2** |
|  | **RR G3-G4/NE : 3,5** | **RR G3-G4/NE : 4,0** | **RR G3-G4/NE : 4,5** |
|  | **In-hospital mortality rate _15-34 Y_: 0,4%** | **In-hospital mortality rate _15-34 Y_: 0,6%** | **In-hospital mortality rate _15-34 Y_: 0,8%** |
|  | **In-hospital mortality rate _35-49 Y_ : 1,1%** | **In-hospital mortality rate _35-49 Y_ : 1,33%** | **In-hospital mortality rate _35-49 Y_ : 1,6%** |
|  | **Premature mortality rate : 1,8%** | **Premature mortality rate : 2,3%** | **Premature mortality rate : 2,8%** |
| VTE | 1762 | 2497 | 3356 |
| In-hospital deaths | 4 | 8 | 13 |
| Premature deaths | 11 | 19 | 31 |

* Number of cases estimated within each five year age group, pooled results

** Data from table 1 of the core text

*** NE: non exposed women

Table S12 - Appendix S3: Number of venous thromboembolic events (VTE) and related deaths attributable to the use of G1 + G2 combined oral contraceptives, results from combined sensitivity analyses

| **Estimated number of attributable cases *** | **‘Low risk’ hypothesis** | ***Working hypothesis*** | **‘High risk’ hypothesis** |
| --- | --- | --- | --- |
|  | **Lower limits of the 95% CI of IR from Lidegaard **** | ***IR from Lidegaard*** | **Upper limits of the 95% CI of IR from Lidegaard **** |
|  | **RR G1-G2/NE** : 1,8** | **RR G1-G2/NE : 2,0** | **RR G1-G2/NE : 2,2** |
|  | **RR G3-G4/NE : 3,5** | **RR G3-G4/NE : 4,0** | **RR G3-G4/NE : 4,5** |
|  | **In-hospital mortality rate _15-34 Y_: 0,4%** | **In-hospital mortality rate _15-34 Y_: 0,6%** | **In-hospital mortality rate _15-34 Y_: 0,8%** |
|  | **In-hospital mortality rate _35-49 Y_ : 1,1%** | **In-hospital mortality rate _35-49 Y_ : 1,33%** | **In-hospital mortality rate _35-49 Y_ : 1,6%** |
|  | **Premature mortality rate : 1,8%** | **Premature mortality rate : 2,3%** | **Premature mortality rate : 2,8%** |
| VTE | 458 | 666 | 911 |
| In-hospital deaths | 1 | 2 | 4 |
| Premature deaths | 3 | 5 | 9 |

* Number of cases estimated within each five year age group, pooled results

** Data from table 1 of the core text

*** NE: non exposed women

Table S13 - Appendix S3: Number of venous thromboembolic events (VTE) and related deaths attributable to the use of G3 + G4 combined oral contraceptives, results from combined sensitivity analyses

| **Estimated number of attributable cases *** | **‘Low risk’ hypothesis** | ***Working hypothesis*** | **‘High risk’ hypothesis** |
| --- | --- | --- | --- |
|  | **Lower limits of the 95% CI of IR from Lidegaard **** | ***IR from Lidegaard*** | **Upper limits of the 95% CI of IR from Lidegaard **** |
|  | **RR G1-G2/NE** : 1,8** | **RR G1-G2/NE : 2,0** | **RR G1-G2/NE : 2,2** |
|  | **RR G3-G4/NE : 3,5** | **RR G3-G4/NE : 4,0** | **RR G3-G4/NE : 4,5** |
|  | **In-hospital mortality rate _15-34 Y_: 0,4%** | **In-hospital mortality rate _15-34 Y_: 0,6%** | **In-hospital mortality rate _15-34 Y_: 0,8%** |
|  | **In-hospital mortality rate _35-49 Y_ : 1,1%** | **In-hospital mortality rate _35-49 Y_ : 1,33%** | **In-hospital mortality rate _35-49 Y_ : 1,6%** |
|  | **Premature mortality rate : 1,8%** | **Premature mortality rate : 2,3%** | **Premature mortality rate : 2,8%** |
| VTE | 1304 | 1831 | 2245 |
| In-hospital deaths | 3 | 6 | 9 |
| Premature deaths | 8 | 14 | 23 |

* Number of cases estimated within each five year age group, pooled results

** Data from table 1 of the core text

*** NE: non exposed women

Table S14 - Appendix S3: Number of venous thromboembolic events (VTE) and related deaths attributable to the use of G3 + G4 combined oral contraceptives (compared to G1 + G2), results from combined sensitivity analyses

| **Estimated number of attributable cases *** | **‘Low risk’ hypothesis** | ***Working hypothesis*** | **‘High risk’ hypothesis** |
| --- | --- | --- | --- |
|  | **Lower limits of the 95% CI of IR from Lidegaard **** | ***IR from Lidegaard*** | **Upper limits of the 95% CI of IR from Lidegaard **** |
|  | **RR G1-G2/NE** : 1,8** | **RR G1-G2/NE : 2,0** | **RR G1-G2/NE : 2,2** |
|  | **RR G3-G4/NE : 3,5** | **RR G3-G4/NE : 4,0** | **RR G3-G4/NE : 4,5** |
|  | **In-hospital mortality rate _15-34 Y_: 0,4%** | **In-hospital mortality rate _15-34 Y_: 0,6%** | **In-hospital mortality rate _15-34 Y_: 0,8%** |
|  | **In-hospital mortality rate _35-49 Y_ : 1,1%** | **In-hospital mortality rate _35-49 Y_ : 1,33%** | **In-hospital mortality rate _35-49 Y_ : 1,6%** |
|  | **Premature mortality rate : 1,8%** | **Premature mortality rate : 2,3%** | **Premature mortality rate : 2,8%** |
| VTE | 887 | 1220 | 1607 |
| In-hospital deaths | 2 | 3 | 6 |
| Premature deaths | 5 | 9 | 15 |

* Number of cases estimated within each five year age group, pooled results

** Data from table 1 of the core text

*** NE: non exposed women
